# Necroscopic Examination of a Case of Hydrocephalus, with Hypertrophy of the Wall of the Cranium

**Published:** 1849-05

**Authors:** E. Mason

**Affiliations:** Wetumpka, Alabama


*Necroscopic Examination of a Case of Hydrocephalus, with hypertrophy of the wall of the Cranium.* By E. Mason, M. D., of Wetumpka, Alabama.

Black, male—æt. 12 years—post mortem ten hours after death. The following table shows the thickness of the bones that were divided in the examination.

| | | |
|---|---|---|
| Frontal, | - - - | $1\frac{1}{8}$ inches. |
| Occipital, | - - - | $1\frac{3}{8}$ " |
| Parietal, | - - - | 1 " |
| Temporal, | - - - | $\frac{1}{2}$ " |

The cellular structure, composing the middle table of the wall of the cranium, was filled with coagulated blood; the internal table was in some places entirely destroyed.

The meninges of the brain were so closely adherent that they could not be separated, and there were no blood vessels traceable on them.

Substance of the cerebrum was of a natural consistence, but preternaturally white; indeed it seemed entirely bloodless.

Convolutions were much separated by the effusion; the character of the fluid presented nothing different from that of serous effusions in general.

Lateral ventricles considerably enlarged by the effusion. Cerebellum softened and of a much lighter colour than natural.

*Remarks.*

We have been unable to obtain anything like a satisfactory history of the case, and can, therefore, only furnish the following imperfect items:

The boy's head commenced enlarging when he was about four years old; whether this was the sequence of any other disease, or under what circumstances it made its appearance, we are totally ignorant. At this time he was attacked with what his mother terms "fainty fits," and his mind was much affected. He, however, gradually recovered his intellectual faculties, but the fits continued at indefinite intervals. We saw the patient for the first time about a month since, and the following were the prominent symptoms: Eyes projected and pupils much dilated; pulse frequent, weak, and wirey; skin warm and dry; he complained almost constantly of a dull aching about the head, but no acute pain; face, hands, and feet would frequently swell towards evening, and go down by the following morning; diarrhœa and occasional vomiting; tympanitis. The "fainty fits" before mentioned, increased in frequency and severity as the fatal issue approached.

To the Editors of the Medical Examiner:

Gentlemen,—I send you the notes, taken by me at the time, of a few cases of Lithontripsy operated upon by the late lamented Dr. Randolph. I was present at all the operations, and kept notes at the time for Dr. Randolph, at his request, who intended to publish these, with many other of his cases of the same kind, in a complete form, with an essay on this mode of operating; his great success rendering it a favourite one with him, as you are probably aware. If you think them of sufficient interest for your journal, in their present condition, they are at your service.

Respectfully yours,

J. M. Wallace.

Philadelphia, April 16th, 1849.

Case I.—*Female child, aged four years, cured in six weeks. Seven operations*

Mary ——, aged four years; general health good; has suffered for several months, from pain in passing water; violent straining, and the usual symptoms of calculus; upon sounding, the stone was felt at once, and after introducing a sound for a few days, to accustom the urethra and bladder to the presence of instruments, a small-sized Jacobson's instrument was introduced to-day, January 22, 1838, but, in consequence of the stone being forced down so close to the internal orifice of the urethra, the expanded part of the blades were beyond it, and the stone could not be seized. It was withdrawn, and a large sized instrument of Heurteloup's used, by which the stone was caught immediately and crushed; caught a second time and again crushed; several small pieces came out in the teeth of the instrument upon its removal. The child struggled violently, but after the operation said that she suffered but little pain.

23d. Passed this morning a number of small fragments, amounting altogether to the size of a large pea. In the evening complained of considerable pain in the bladder; ordered a hip-bath.

27th. Passed a large fragment this morning. Since the last report has been very well, and playing about the room, complaining only when voiding her urine.

28th. Caught the stone this morning, the blades of the instrument being separated *an inch and a quarter* when it was grasped. After crushing it, three fragments were caught and crushed; a slight discharge of blood followed the removal of the instrument.

Feb. 3d. For two days after the last operation, she passed several small fragments; nothing has come away since that time. The sound showed still the presence of several pieces of stone. She now retains her water for several hours.

Feb. 4th. Stone caught four times to-day, the blades of the instrument being expanded from a fourth to a half an inch; some blood followed the operation; but she suffered so little that she brought her doll with her to the table, and when led away, insisted on coming back for it and remained quietly, talking with us for several minutes.

8th. Complained of great pain in voiding the urine to-day. Ordered the hip-bath.

9th. Some fever during the night; tongue covered with a white fur; ordered a dose of magnesia.

11th. Appears to be very well; stone caught three times; instrument expanding one inch, a half an inch, and a fourth of an inch.

18th. Has passed several fragments since the last report, and a quantity of sand. Stone caught four times to-day.

20th. A large sound introduced in order to dilate the urethra.

25th. Passed three fragments the size of small peas since last report; four small pieces were caught to-day and crushed.

26th. Passed a tea-spoon full of very small fragments since the last report; stone caught twice to-day.

March 10th. Has passed several pieces since the 26th; *one* very large one; has been sounded very carefully, and nothing is to be felt in the bladder; her general health is excellent, and she retains her water for many hours at a time, and when voiding it has no pain or inconvenience whatever.

April 25th. This patient was heard from to-day, and has continued perfectly well ever since.

During the whole course of treatment this little girl was allowed to run about her room, and was never confined to bed, except for one day, when she had a little fever, as mentioned on Feb. 9th. It will be seen, moreover, that the stone was of considerable size. At no time was any operation continued longer than three minutes.

CASE II.—*Male, aged 21 years; cured in six weeks; twelve operations.*

Levi K., aged 21 years; good general health; has suffered for nine months from symptoms of stone. He was sounded in the country by a surgeon, who readily detected the stone, and advised the operation of lithotomy. His brother, who is a physician near Lancaster, brought him to Philadelphia, and placed him under the care of Dr. Randolph. He suffers but little, and has so little irritability of the bladder that he can retain his urine all night.

Sept. 28th, 1840. After remaining here for a week, in the presence of Drs. Horner, Henderson, his brother, and myself, Dr. Randolph caught the stone, with a large sized Jacobson's instrument, twice, and crushed it; the blades were open the first time to their full extent, when grasping the stone. The patient said that the operation was not more painful than the sounding to which he had previously submitted. Ordered twenty drops of laudanum, and to drink freely of flaxseed tea, with a small portion of sweet spirits of nitre in it, and to be confined to a vegetable diet.

29th. At 4 o'clock, P. M., had a severe chill, followed by fever; ordered a hip-bath, and the application of bags of hot sand to the pubes.

30th. Is to-day as well as usual.

October 3d. Has passed three fragments and a small quantity of sand since yesterday. The stone was caught and crushed five times to-day. Made no complaint of pain.

Oct. 7th. Present Drs. Ruan, Jackson, Page, and M'Pheeters. The stone was caught three times to-day, and was so hard that it was necessary to wrap the screw with a towel, in order to be able to use sufficient force to break it.

Oct. 8th. Passed a round black portion, as large as a pea, which appears to be the nucleus of the stone; around it there is a thin white layer, an analysis of which proves it to be the oxalate of lime covered by the phosphate.

Oct. 11th. Has passed three fragments; stone caught four times. He has passed but little stone, although it has now been broken fourteen times altogether.

Oct. 15th. Caught the stone three times to-day.

Oct. 19th. Several large pieces of stone felt just at the neck of the bladder, which, probably by blocking up the urethra, prevent the smaller portions from coming away. To-day, the stone was caught four times, and one piece, judging from the expansion of the instrument, nearly an inch in size.

Oct. 25th. Caught four times. The bladder was very irritable after this operation; he passed his water every three-quarters of an hour, and has had a slight chill followed by fever; ordered laudanum injection and hip-bath. He has discharged a teaspoonful of fragments to-day.

Oct. 29th. Present Drs. C. D. Meigs and Wilson of Berwick. Operated on four times; passed several pieces during the day.

Nov. 5th. Has passed about two teaspoonfuls of fragments, and three large pieces since the last report. The stone was caught four times. He suffers no inconvenience whatever now from the operation, and dresses himself and walks about his room as soon as it is over.

Nov. 8th. Stone caught five times.

Nov. 12th. A large fragment lodged yesterday in the urethra, but was readily pushed back into the bladder by the sound. He passed in the course of a few hours a table-spoonful of fragments.

The stone was caught six times to-day.

Nov. 16th. Has passed a very large quantity since the last report. All that has come away nearly fills a common ounce box. The stone was caught to-day four times.

Nov. 20th. Has continued to pass fragments until yesterday, when he said that there was no more in his bladder, and upon sounding him carefully nothing could be felt, and he was allowed to return home.

This patient sent another one to Dr. Randolph two years after, and was at that time perfectly well. The case was a very interesting one, from the great size and hardness of the stone, and the number of times it was necessary to break it. It will be seen that it was broken no less than *forty eight times* in the twelve operations, and besides the mass of stone which fills an ounce box, a quantity of fine sand was passed which was not collected. The patient had fortunately a very healthy bladder, and consequently suffered but little from the frequent repetitions of the operation. There was a case under treatment at the same time who was cured in but two operations, the details of which are below.

CASE III.—*Male, aged 41 years; cured in two weeks; two operations.*

John D——, a farmer, from Columbia Co., Pennsylvania, six years ago received a severe blow on the back, which was followed by an attack of nephritis. For more than a year he has suffered from symptoms of stone; he has never passed any sand, nor can he recollect having an attack of nephritic colic since his injury. The stone was readily felt on sounding, and thought to be small. After remaining for a few days in town, he was operated on Oct. 29th, in the presence of Drs. C. D. Meigs, and Wilson of Berwick, immediately after the preceding case, and in the same room: the stone was caught three times.

30th. Has had a great deal of scalding in voiding his urine, which was tinged with blood. The violent straining in emptying the bladder has produced severe headache, which he states has frequently occurred from the same cause; ordered laudanum injection and four hours after a dose of castor oil. To drink freely of flaxseed tea with spirits of nitre in it. A-hip bath and warm sand to the pubes, &c.

31st. Passed a very bad night, pulse 90, and quick, tongue furred, complains of excessive scalding in voiding his urine ordered a seidlitz powder, and to repeat the hip-bath and laudanum injection.

Nov. 2d. All his unfavorable symptoms have passed off. Enough stone has been discharged to make, if put together, a mass nearly as large as a pigeon's egg. Says that he feels as if there was no stone left; not sounded yet, from a fear that the irritability of the bladder has not yet subsided.

Nov. 8. Has continued to improve until last night, when he was obliged to get up to empty the bladder, and says he feels something at the neck of the bladder. Upon sounding, a small fragment was felt and broken once.

Nov. 12th. Passed last night a few minute fragments, and upon sounding to-day nothing is felt.

A few days after this he returned home, and I heard from him four months after, when he remained perfectly well.

CASE IV.—*Male child, aged 4 years ; cured in two weeks ; two operations.*

Horace R., aged 4 years, has suffered from symptoms of stone for more than two years.

Oct. 8th. He was examined to-day. Present, Drs. Yardley and Kirkbride. Upon feeling the stone, a small sized instrument of Heurteloup was introduced, and the stone caught and broken three times. He does not retain his water any length of time, and is constantly wet. The stone is very soft, and broke very easily.

Oct. 10th. Is much relieved; has passed several small fragments.

Oct. 14th. The stone was caught once to-day ; he now retains his water, and his mother says " is better than he has been for six months."

Nov. 5th. Has passed a few small pieces and a quantity of sand since the last report ; nothing to be felt in the bladder. I heard from this patient two years after, and he remained perfectly well. This, I believe, was the youngest *male* child ever operated on by Dr. Randolph.

The following case of a child occurred in my own practice:

*Male child, aged 4½ years; cured in three weeks; three operations.*

Pearson H., aged 4½ years, has been sickly from his birth, and suffered much from disorder of the abdominal viscera. Dr. J. F. Meigs, who is the family physician, requested me to see him, Nov. 7th, 1847, as he suspected the presence of a stone in the bladder. Upon sounding, it was thought that a stone could be felt, but his struggles were so violent, and the bladder contracted so forcibly, that we were not certain of it. He was ordered palliatives, with directions to be informed if any change took place in his symptoms.

March 18th, 1848. He has become much worse, and upon sounding him to-day a stone was unequivocally felt. After introducing a sound every day for a week to accustom the parts to the presence of instruments, he was ordered a laudanum injection at 9 o'clock in the morning; and at 11 on the 25th, in the presence of Dr. J. V. Patterson and Dr. E. Wallace, I introduced a small sized instrument of Heurteloup, and caught the stone and broke it *once.* A considerable quantity came out in the teeth of the instrument.

April 1st. Has been very comfortable since the operation, and retains his water for two hours at a time. The operation was repeated to-day, and the stone caught twice and broken readily. It is very soft, and upon analysis found to be composed of uric acid.

April 5th. Present Dr. F. West. Has passed half a teaspoonful of fine sand since the last report. The stone was caught twice to-day and broken.

April. 6th. Complains of pain in the urethra which was relieved by a laudanum injection. All his symptoms disappeared after this operation. He passed some fine sand for a few days. Upon sounding him a week after, the bladder was found perfectly free from any fragments. He soon after went to Cape May for the summer, and his father informed me a few weeks since that he remained perfectly well, and had had no return of his complaint since. As a matter of prudence I directed him to remain in bed after the operation, but he could not be persuaded to do so, and was allowed to be about his room, and during the treatment went about the house as usual.

